# Reporting guidelines on remotely collected electronic mood data in mood disorder (eMOOD)—recommendations

**DOI:** 10.1038/s41398-019-0484-8

**Published:** 2019-06-07

**Authors:** Maria Faurholt-Jepsen, John R. Geddes, Guy M. Goodwin, Michael Bauer, Anne Duffy, Lars Vedel Kessing, Kate Saunders

**Affiliations:** 1 The Copenhagen Affective Disorder Research Center (CADIC), Psychiatric Center Copenhagen, Rigshospitalet, Copenhagen Denmark; 2 Department of Psychiatry, University of Oxford, Warneford Hospital, Oxford, UK; 3 Oxford Health NHS Foundation Trust, Warneford Hospital, Oxford, UK; 4 Department of Psychiatry and Psychotherapy, Carl Gustav Carus University Hospital, Technische Universität Dresden, Dresden, Germany; 5 Department of Psychiatry, Queen’s University, Kingston, ON Canada

**Keywords:** Psychiatric disorders, Bipolar disorder

## Abstract

Prospective monitoring of mood was started by Kraepelin who made and recorded frequent observations of his patients. During the last decade, the number of research studies using remotely collected electronic mood data has increased markedly. However, standardized measures and methods to collect, analyze and report electronic mood data are lacking. To get better understanding of the nature, correlates and implications of mood and mood instability, and to standardize this process, we propose guidelines for reporting of electronic mood data (eMOOD). This paper provides an overview of remotely collected electronic mood data in mood disorders and discusses why standardized reporting is necessary to evaluate and inform mood research in Psychiatry. Adherence to these guidelines will improve interpretation, reproducibility and future meta-analyses of mood monitoring in mood disorder research.

## Remotely collected mood data in mood disorder research

Mood disorders are among the most commonly diagnosed mental disorders. Unipolar disorder affects approximately 1 in 4 people over a lifetime^1^. Bipolar disorder, characterized by recurrent episodes of sad/depressive and elated/manic mood, has an estimated prevalence of 1–2% and is a major cause of morbidity and mortality^2,3^. The changes in mood that characterize unipolar disorder and bipolar disorder are accompanied by shifts in cognitive function, energy, activity, sleep and other behavioral aspects that may be quantified^4–7^.

In unipolar and bipolar disorder, mood deviations present both in the form of a mood episode and in the form of sub-syndromal mood instability, and mood instability has been suggested as a part of the bipolar prodrome^1,8,9^. During the last decade there has been a gradual shift of therapeutic focus from mood episodes to inter-episodic mood instability^10–12^. Unlike mood state which describes a more prolonged prevailing state or disposition^13^, mood instability may be reflected by changes in polarity and severity of mood deviations as well as by the frequency of shifts in mood on a more daily or weekly basis. A substantial proportion of patients with mood disorders experience mood swings on a daily basis, and instability of mood has clinical significance in and of itself. Frequent monitoring on a daily or weekly basis may also capture the reactivity of mood (often termed affect), whereas less frequent monitoring is more likely to capture only the average mood state. Reactivity of mood reflects response to environmental situations or events. It can be both over or under-reactive, for example Kraepelin’s description of depressive patients as insensitive to bad news. Both mood reactivity and mood states are altered in patients with unipolar and bipolar disorder, as well as in those at identifiable high-risk^14^. Thus, mood instability is part of the prodrome of both unipolar disorder and bipolar disorder, and has been associated with substantial disability including impaired functioning, increased risk of hospitalization, high risk of relapse, and substance misuse in bipolar disorder^8,9,12,15–21^. Due to the clinical importance of mood instability, it has been suggested as a target for treatment in its own right. Mood instability could prove to be a more sensitive measure of outcome in randomized controlled trials (RCT) than remission, relapse or recurrence of full-blown mood episodes^8,22–24^, and would make meaningful early phase proof of concept trials more efficient for both drug and psychological interventions.

Consecutive monitoring of mood symptoms and episodes dates back to Kraepelin who made frequent observations of the prospective clinical course of his patients^25^. Patient-based self-reports evolved in the context of structured psychological treatments, when the first controlled treatment trials were conducted in the 1960s. Self-monitoring has come to be viewed as a helpful component of cognitive behavioural therapy, dialectical behavioural therapy and in patient self-management of chronic diseases generally. Thus, frequent, or even continuous, fine-grained measurements of mood (i.e., polarity, stability, context) in clinical, high-risk and epidemiological populations provides an important opportunity to gain a better understanding of the nature, correlates and clinical implications of mood disorder.

Today self-report measures are ubiquitous in psychiatric research, and various mood charting instruments for self-monitoring have been used in the management of mood disorders in clinical samples. Paper-based mood charting instruments, such as the National Institute of Mental Health LifeChart Method (NIMH-LCM)^26^ and the Systematic Treatment Enhancement Program for Bipolar Disorder (STEP-BP), have proven validity compared to clinical rating scales for depression and mania^27,28^. Paper-based mood charting instruments can be viewed as facilitating tools helping patients gain illness insight, facilitate patient empowerment, teach patients to recognize early warning signs of recurrence of mood episodes and enable individualized characterization of mood and mood instability in detail. However, paper-based mood charting is potentially inconvenient, time consuming, costly and unreliably time stamped. These limitations lead to low patient-compliance, and potential recall bias when reporting data retrospectively, i.e., where patients complete batches of daily ratings at a single time point (sometimes referred to as *hoarding* or *backfilling*)^29–33^. Retrospective recall bias may be a particular problem for mood monitoring because patients need to recall both variation and intensity around a global mean^24^.

Digital technologies that are widely accepted by the general public are being integrated into the routine care of bipolar disorder to increase patient involvement and expand clinician oversight between visits. They provide suitable platforms for active or passive patient monitoring including computers and smartphones. Many applications are available today to monitor bipolar disorder away from medical settings that require active patient participation. These include validated products for daily mood charting such as the ChronoRecord (www.chronorecord.org) on a computer^33^, the MONARCA system on a smartphone^34^, and the Life-Chart on a smartphone and web site, the use of text messaging, e-mail and web-based entry implemented for weekly self-report of mood scores (e.g., by the True Colours programme (^8^; https://oxfordhealth.truecolours.nhs.uk/www/en/), and weekly or monthly use of an interactive voice response (IVR) system to complete the PHQ-9. In all cases, the patients are promoted to respond to questions about symptoms of their illness. In addition to self-reported symptoms information about parameters such as daily medications taken, activities done during the day, sleep (time, quality etc.) can be collected long-term detailed characterization and research. Although challenges remain regarding the interpretation of self-reported data, much of the current knowledge on the psychopathology of bipolar disorder, particularly in regards to the quality of remission between acute episodes, has been informed by the daily recording efforts of patients worldwide.

Digital electronic and remote self-monitoring of mood offers the possibility of ecological momentary assessments (EMA)^35^ for remote fine-grained assessment in real-time and in naturalistic settings. It allows the timing and compliance of data collection to be verified and eliminates the need for costly and error-prone data entry. The facility to send prompts to complete ratings may help remind patients to perform the self-monitoring and may have higher utility (feasibility) and lower intrusiveness compared to the paper-based alternatives. The pattern of responding to prompts also provides an informative data stream. In the Cequel trial the time taken to respond to prompts was found to predict improvement in depressive symptoms in advance of any change in mood score^36^. Furthermore, remote self-monitoring methodology is a way to aid individuals to gain greater insight into the dynamic and temporal nature of mood and mood stability in mood disorder in daily life^11,37^.

The rapid evolution of smartphone technology and the ubiquity of mobile networks have seen increasing growth of e-mental health technologies^38^. These include electronic platforms offering tools for remote self-monitoring of mood and smartphone apps for self-monitoring of mood and other symptoms related to mood disorders. These have been implemented and used by several researchers in observational studies and randomized controlled trials^34,39–42^, and enable collection of data on daily mood and sub-syndromal mood fluctuations.

Today there is a particular abundance of publicly and commercially available applications for monitoring mood. While these appear to be popular among consumers, significant concerns have been raised about their quality and evidence base^43^. In a review from 2015, Nicholas et al. reported that none of the symptom monitoring apps that they identified had a duty of care alert, few had been subject to rigorous research evaluation, or cited published material about the app, some provided wrong information regarding the illness. Only a small proportion had a privacy policy. Further, the lack of rigorous research evaluation of the potential benefits and pitfalls with smartphone-based monitoring and treatment has been emphasized^43^.

Within research settings much greater care has been taken to embrace some of these issues. The MONARCA studies showed that the self-reported severity of depressive and manic symptoms by patients with bipolar disorder correlated well with clinically rated symptoms measured using the Hamilton Depression Rating Scale and the Young Mania Rating Scale^39,44,45^. However, overall there was no immediate benefit of smartphone-based monitoring on the severity of depressive and manic symptoms in these patients. Another study confirmed that smartphones have the potential to monitor bipolar disorder symptoms in daily life^46^. RCTs investigating the potential positive as well as negative effects of smartphone-based treatment in mood disorders are ongoing^34,40,47^. The use of objective smartphone-based sensor data to reflect illness activity and bipolar disorder diagnosis have also been conducted^41,44,48,49^. Further, remote capture monitoring of electronic mood data in high-risk groups such as the adolescent and emerging adult offspring of bipolar or depressed parents may be a fruitful new direction of early identification and prevention research^50^.

The holy grail would be a completely frictionless measure using a wearable device linked to a smartphone or from data from the smartphone itself, that would accurately estimate or predict mental state. This might be possible from geolocation signals, bodily movement, the speed and accuracy of keyboard use, patterns of text and phone use or voice quality.

Overall, few of these research approaches have been developed for wider public use. Moreover, native mobile phone apps require users to download them in the first place, need ongoing updating as operating systems change and may run down the smartphone’s battery. While there are moves towards the use of mobile websites, which can be used across devices (i.e., smartphone types, iPads etc.), it currently remains difficult to link these with other objective smartphone-based sensor data.

Despite the rapid expansion in their use standardized measures and methods to collect, analyze and report electronic mood data are lacking. Therefore, this paper aims to provide an overview of the status of remotely collected electronic mood data in mood disorders research and make the case why standardized reporting is necessary to advance research into the nature and course of mood disorders, inform clinical practice, and ultimately improve outcomes for individuals at risk of or suffering from mood disorders. Following from this overview, we propose guidelines for electronic mood monitoring research (eMOOD). Adherence to these guidelines will improve interpretation, reproducibility and future meta-analyses of mood data from independent research studies.

## Drawbacks of inconsistent study reporting and lack of consensus

Based on a literature search covering mood disorders and electronically measured mood conducted in PubMed, PsychInfo and Embase, the studies that were identified had drawbacks in each of the individual original studies, and these were used to suggest a minimum of guidelines for reporting of electronic mood data to improve the design, analysis and reporting of future studies (Table 1).

**Table 1:** eMOOD: guidelines for reporting on remotely collected electronic mood data in mood disorder

Topic	Checklist item
*Participants*	
Study group selection	Recruitment details; Diagnostic assessment; Illness severity assessment; experience level of assessor
Comparison group selection	Recruitment details; Methods used to rule out psychiatric illness
Participation rate	Detailed description on participation rate of participants versus non-participants
Inclusion and exclusion criteria	Detailed description of inclusion and exclusion criteria
Disease characteristics	Age at first depressive episode, age at first hypomanic/manic episode; duration of illness; severity of illness; comorbidities; medication use; substance misuse; number of previous mood episodes
Demographics	Age; sex, socioeconomic status; education
Study design	Time of baseline assessments, follow-up period, blinding of assessors
*Data collection*	
Hardware / software	Software; online; app-based; software available for public or not; participant level of technical skills; funding of system used; investigator or industry-initiated study
Questionnaires/patient-reports used	Description of the self-report measure(s) used; dimensionality validation in the participant group
Collection details	How often are participants asked to complete mood ratings; time of day; Instructions; whether retrospective entering was possible
*Data analysis*	
Missing data	% of missing data; imputation method employed; criteria for participant exclusion on basis of missing data; comparison of those excluded with included participants (see Table 2 for more details)
Method of analyses	Analyses planned in advance with clear definition of the primary, secondary and tertiary analyses/outcome measures including definition of covariates for adjustment (protocol); Metrics used; software/script employed in the calculations; transformations if used; unadjusted and adjusted models reported
Conflict of interest	All potential conflicts of interest clearly stated

Inconsistent reporting of research methods, analyses and results impedes the assessment of studies. Readers need to be able to assess the strengths and weaknesses of the entire study methodology and have these presented in the context of the wider field. Failure to report necessary details can significantly hamper efforts to make comparisons including calculation of summary effect sizes between studies in future meta-analyses and replication studies. Published research also influences subsequent design of new studies such that greater methodological rigour and standardization assists future authors in experimental design and data collection. In addition, more consistent reporting of studies may speed up the peer review process.

### Questionnaires for electronic self-monitoring of mood

Even in studies where the experimental design is clearly reported the absence of any agreed points of reference can still make study comparison challenging. For example the daily monitoring used in the AMoSS study^51^, conducted by some of the authors of the present paper, was a 6 item mood questionnaire (anxious, elated, sad, angry, irritable, energetic—all scored on a Likert scale of 1–7) anchored to weekly self-reported clinical symptoms on the Quick Inventory of Depressive Symptomatology (QIDS) and the Altman Self-rating Scale for Mania (ARSM). On the other hand the MONARCA studies^39,41,42^, conducted by other authors of the present paper, used a single scale to evaluate mood on a daily basis (scored from depressive to manic on a scale from −3, −2, −1, −0.5, 0, + 0.5, + 1, + 2, + 3) anchored to clinician evaluated depressive and manic symptoms collected fortnightly using the Hamilton Depression Rating Scale (HDRS) and the Young Mania Rating Scale (YMRS). While there are equivalent scores for example for the QIDS and the HDRS, no direct comparisons of the daily measures can be made. Furthermore, the question of the clinical status of the patient is an additional issue as frequent contact can act as a form of intervention and thus alter the outcome measures of interest. While validated rating scales are reported to have good reliability and reproducibility, these are often for use in very specific patient groups and their generalisability to those with other diagnoses and comorbidities is unclear.

### Adherence to electronic self-monitoring

Adherence to electronic self-monitoring is an important factor to consider and report as is often low and can have a significant impact upon the reliability and representative nature of the data. If significant levels of missing values are present, a detailed description on how these were handled should be included so that readers and future studies have the possibility to assess and replicate the methods used. The nature of smartphone data, and particularly phone sensor data, complicates data sharing and few groups currently make their data available for secondary analyses or to facilitate combining datasets.

### Analyses of mood data

Mood data can be quantified in many different ways and is further complicated by the lack of consensus about how mood and specifically mood instability is defined, measured and reported^18,21,24,52^. The nomenclature includes intensity, valence, entropy, reactivity, variability, frequency of mood changes, and affective phase, all of which require different methods of quantification and represent different dimensions of mood states.

Overall, standardized measures and methods to collect, analyze and report remotely collected electronic mood data are lacking. With the present paper, we propose below a set of minimum standards for reporting such data (eMOOD) (Table 1). These could assist researchers in reporting their mood data, assist readers in how to critically examine the existing literature on mood reporting in mood disorders, aid the reproducibility and production of meta-analyses within the field, and ultimately provide insights into the pathophysiology of mood disorders.

## Guidelines for reporting on remotely collected electronic mood data—eMOOD

To help and standardize reporting of remotely electronically collected mood data, we propose the mood reporting guidelines: eMOOD (Table 1). Adherence to these guidelines will help improve interpretation, reproducibility and future secondary analyses and meta-analyses of mood and mood instability in mood disorders.

### Participants (top section of Table 1)

#### Study group selection

The description of participant groups is an essential, but often under-reported, feature of any scientific paper. Details on where, when and how the recruitment occurred should be specified—for example through advertisement or subspecialty clinical program. Especially for case-control studies this information is crucial—including details on excluded participants and main reasons for exclusion. The method for determining eligibility including diagnosis (or in high-risk research diagnosis of the parent or relative) should be described as should the experience of the person administering the diagnostic assessment or interview. Furthermore, details on which diagnostic system (the Diagnostic and Statistical Manual for Mental Disorders or the International Classification of Disease) was used should be specified as these differ. Psychiatric classification can be challenging and the rigid application of diagnostic criteria, especially from structured interviews conducted by trained but clinically inexperienced raters (and independent of the context in which they occur) may generate misleading diagnoses. On the other had self-report diagnostic instruments are variable, even idiosyncratic in nature and while some show reasonable consistency with clinician-based assessments they can make comparisons between studies difficult.

#### Comparison group selection

Recruitment of a proper comparison group, especially in case-control studies, can be difficult. Often studies recruit as controls healthy individuals from a selected population not necessarily from the same population as the patients or high-risk individuals, e.g., family members of patients, introducing possible confounding variables. Further, controls may only differ from the study population by specific diagnosis (i.e., both clinical populations) or consist of ‘super-healthy’ individuals not representative of the general population^53^. Recruitment via advertisement or the internet may result in selected populations not representative of the target population or the general population and the recruitment via national registers, as possible in some countries, may lead to low participation rates. Future studies should critically take initiatives to minimize selection bias increasing the validity and generalizability of study results. Regarding the statistical analyses comparing cases and controls, unadjusted analyses as well as analyses adjusted for possible confounding variables should be reported so that it is possible to interpret whether the differences between groups reported reflect true findings.

The increasing availability of polygenic risk scores may provide an interesting way of estimating predisposition to psychiatric disorder on the one hand and resilience on the other in a range of populations, most notably young people. The more fine grain the phenotype the more potential there may be for useful correlation with genetic data. Mendelian randomization may offer a technique for testing specific causal hypotheses about variety of exposures and genetic risks^54^.

#### Participation rate and analyses of participants versus non-participants

The participation rate and analyses of participants versus non-participants are rarely reported in studies of remotely collected mood data. Participants in such studies may be younger, more well educated and suffer from less severe mood disorders than non-participants, and characteristics of participants may differ for patients and individuals included in the control group. Such differences may potentially result in selection bias and decrease generalizability of findings.

#### Inclusion criteria

Inclusion and exclusion criteria should be explicitly stated. Sex, age, socioeconomic level, psychiatric and somatic comorbidity, alcohol and recreational drug use, psychotropic treatment, including antidepressant use, are all known to influence mood states, and all should be in any protocol.

#### Disease characteristics

Mood disorders are highly heterogenous in terms of etiology and phenomenology – often including contradictory symptoms contained within a single disorder (e.g., both hypersomnia and insomnia form part of the criteria for a depressive episode). Also, age of onset, sex and treatment response are known to be associated with differential clinical trajectories^55–57^. For example, early age of onset and sex have been associated with a more chronic clinical course with higher levels of mood instability^19^. Furthermore, an excellent response to long-term lithium prophylaxis identifies a more homogeneous subtype of bipolar disorder similar to classical manic-depressive illness and characterized by a highly recurrent episodic course with good quality of remission^58^. This means that diagnostic specifiers should be described where possible. In addition, mood state at study entry should be stated as well as illness duration, number and polarity of previous mood episodes and inter-episode functioning.

#### Demographics

Mood instability has been suggested to decline with age, to be more common in women than men, and more frequently reported in those who are in part-time employment and those on lower incomes^59–61^. As a minimum, these variables should be considered as possible covariates in the statistical analyses.

#### Study design

Details on study design including if possible access to a study protocol or link to registry (https://www.researchregistry.com; https://clinicaltrials.gov) prepared in advance specifying the statistical analyses (see methods of analyses below), cofounding variables to include in statistical analyses, outcome measures and power calculations.

Clearly stated data on mood and clinically assessed symptoms collected at baseline should be included together with the duration of the study and information on frequency and total number of clinical evaluations.

### Data collection (middle section of Table 1)

#### Hardware/ software

Some monitoring systems have only been developed for specific operating systems (Android or iOS), and this dictate other functionalities linked to the specific type of smartphone. Details of smartphones models used in studies should be included so that readers can clearly assess possible differences between studies.

The way in which questionnaires/patient-reported mood scales are delivered to patients has a significant impact on response rates^62,63^. The use of prompts and the algorithms employed for repeat prompts are also pertinent here. Capability to amend results should be stated as should the way in which patients are able to visualize their data: there is preliminary evidence that mood monitoring per se may be considered an intervention of sorts^64^ although current data suggest that continuous monitoring of mood (and other variables) has no beneficial or detrimental effects on mood^39,45^. Data storage arrangement including security should be stated as should any on device processing.

#### Questionnaires/patient-reports used

The choice of mood measure is also an important consideration in study design. There are a wide range of validated mood questionnaires available, although many are only validated for use in specific populations. While most research studies employ validated scales, publicly available apps tend not to do so. Validated scales are preferable scientifically, but the move towards app-based monitoring poses a challenge as the majority of these tools are lengthy and unwieldy to answer on a smartphone screen. A wide range of approaches have been employed in publicly available apps including single mood scales with depression and mania as two extreme poles or anchor points, emotion icons or ‘emoticons', and pre-selected mood states. Each study should state the method for measuring mood used, rationale and the validity optimally in relation to a well validated observer-based blinded assessment of mood^41,44^ in their study population.

#### Collection details

The frequency with which mood is reported and the study population being targeted varies widely between studies. The majority of systems employ weekly ratings using validated measures such as the QIDS or the ASRM, but more frequent (i.e., daily) ratings using shorter mood reports are commonly used and are often preferred by patients. More frequent monitoring may also capture the reactivity of mood (often termed affect), whereas less frequent monitoring is more likely to capture the average mood state only. The important issue is how and what patients or study participants are instructed to evaluate, and therefore what the data actually measure. Each study should clearly state how the participants were instructed and educated to evaluate their mood state.

Reactivity of mood reflects response to environmental situations or events. It can be both over- or under- reactive, for example Kraepelin’s description of depressive patients as insensitive to bad news. Mood state reflects a more prolonged prevailing state or disposition^13^. Both mood reactivity and mood states are altered in patients with unipolar and bipolar disorder, as well as in those at identifiable high-risk^14^ and as such may warrant prospective monitoring. The ideal frequency of mood reporting may also differ between high-risk or diagnostic groups and for different outcomes (i.e., prediction of onset, recurrence, monitoring of the quality of remission or psychosocial functioning). For example, individuals with borderline personality disorder report very frequent changes in affect (multiple times per day) which are likely to be missed using weekly monitoring, whereas someone with euthymic bipolar disorder will likely need a lower frequency of monitoring. Preliminary data suggests that in youth at confirmed familial high-risk for bipolar disorder, changes in temperament and increased variability in weekly mood ratings maybe correlates of the early stages of emerging illness^50,65^. Given that mood instability is a common experience across mental illnesses, details on frequency of reporting are important for interpretation of results.

### Data analysis (bottom section of Table 1)

#### Missing data

Missing data is ubiquitous in self-reported mood data and poses a significant methodological challenge. There are many causes of missing data and their understanding can assist in deciding how best handle this in analyses (Table 2). Mood data may be missing at random (i.e., unrelated to the mood state) or not at random (i.e., missing because of mood state)^66^. For the latter, any attempts to impute the missing data may inadvertently lead to the loss of meaningful data. Replacing missing data with the mean or median does not take into account the time series nature of the data and makes no assumption about the relationship between variables: it has the overall effect of reducing the variance in the dataset. This approach also risks weakening covariance and correlations in the data. Last observation carried forward or next observation carried back are common statistical approaches to the imputation of missing data in time series; both can introduce error if the data has a clear trend and make assumptions that might not reflect the true mood state of that particular patient. Linear interpolation is preferable where there is a clear trend, but not if data is oscillatory which often is the case of mood disorders. Regression substitution can be used to estimate missing values but can overestimate model fit and reduces the variance. Maximum likelihood estimation identifies a set of values that are most likely to have resulted in the observed data using all available data to calculate a log likelihood. Multiple imputation is a more sophisticated approach which utilizes correlations between data and creates values for the missing data based upon these correlations: it then averages the simulated data by incorporating random errors in the prediction. This approach provides more accurate variability, as it considers variability due to sampling and due to imputation; however, it can be complex to employ. Alternative approaches where missing data is viewed as adding dimensionality are also being developed. Finally, if there is too little data to include then participants may need to be excluded from the analysis. This can limit the generalizability of the findings as poor responders may represent a particular phenotype or state of illness severity. Given the impact on the final results of missing data, it is essential that the strategy for dealing with it is explicitly stated and rationalized.

**Table 2:** Approaches to handling missing data

Method	Description	Limitations
Replacement with mean or median	Inserts the mean or median of the whole dataset in place of missing data	Reduces the variance
Last observation carried forward/back	Inserts the last observation in place of the missing data points	Ignores existing trends in the data
Reduces variance		
Weakens covariance and correlations		
Linear interpolation	Assumes a linear relationship between two points and uses non-missing values from adjacent points to compute a value for the missing data points	Inappropriate in oscillatory data
Regression substitution	Predicts the most likely value of the missing data	May overestimate model fit
Does not quantify uncertainty about that value		
Reduces variance		
Maximum likelihood estimation	Identified likely set of values based on observed data. The maximum likelihood estimate of a parameter is the value of the parameter that is most likely to have resulted in the observed data	Limited to linear models
Multiple imputation	Plausible values for missing observations are created that reflect uncertainty. These values are used to impute the missing values. This process is repeated, to create a number of ‘completed' datasets. Each of these datasets is separately analyzed. The results are then combined allowing the uncertainty of the imputation to be taken into account	Complex to employ
Choosing the correct model can be difficult		
Dimensionality	Views missing data as an additional dimension within the data	Complex to employ. May be difficult to interpret

#### Methods of analyses

Within mood disorder, mood varies over time in both severity and polarity and these changes can be dramatic and unpredictable. While most clinical trial settings use standard summary statistics to describe mood outcomes (e.g., mean, median) there is increasing interest in how mood instability can be mathematically quantified and how changes over time can be modelled (Table 3). While the phenomenological features of mood disorders provide a starting point for analyses, there are currently no biological systems on which to base the parameters of a model and a series of quite arbitrary assumptions must be made.

**Table 3:** Mathematical approaches to quantifying variability of mood data

Statistical method	Assumptions	Limitations
Time domain e.g., RMSSD	Normally distributed data	Influenced by extreme scores No estimate of the width of the distribution Do not distinguish different signals Examples of datasets with identical means, SDs and RMSSDs with very different underlying data structure
Frequency domain/ Spectral analysis	Data considered a sum of sinusoidal oscillations with distinct frequencies Analyses require stationarity within data	Long data series required
Entropy	Considered a measure of randomness/irregularity Should be calculated on non- normalized time series	Accuracy reduced in short time series Sinusoidal trends are detrimental Spikes in the data can impair linear estimates

In the absence of an agreed semantic definition and reporting method for mood instability^24^, the mathematical quantification of instability has been done in a variety of ways^18,20–22,51,52,67^. Time series analyses are the simplest means of representing variability and these include metrics such the standard deviation (SD) and root means squared successive differences (RMSSD)^68,69^ (Table 3). Standard deviation provides a measure of the variation of a set of values and the extent to which those values deviate from the mean. However, SD is an inappropriate measure of dispersion in skewed data, does not provide any estimate of how far typical values tend to be from the mean and is influenced by extreme scores. RMSSD is a simple algorithmic approach widely used in the quantification of heart rate and heart rate variability. The difficulty with these time domain measures is that they do not reliably distinguish different signals, and there are many examples of datasets with identical means, SDs and RMSSDs with very different underlying data structure.

Frequency domain or spectral analysis is an alternative method for quantifying time series data in which the data is considered as a sum of sinusoidal oscillations with distinct frequencies: the amplitude and phase of the frequencies describe the underlying signal^70^ (Table 3). Frequency domain analyses require stationarity within the data (i.e., that properties such as the mean and the standard deviation of the signal remain constant throughout the recording period); it is often used in the analysis of heart rate variability^69^. More complex explicitly oscillatory models have been also been proposed^22^, but the oscillation periods are either too long to clearly identify periodicity^71,72^, too short or too noisy^22^.

Entropy analysis is a measure of randomness or irregularity within the data and is sometimes used to represent complexity (Table 3). It has the advantage that it can be applied to relatively short series of data (100–900 data points)^73^. The order of the data is important, but once again non-stationary data can compromise meaningful interpretation. Other approaches such as network analysis^74^ and signature-based learning models have also been used to analyse mood data. These focus on the interrelationship between different mood dimensions (e.g., how anxiety, low mood, irritability interact) rather than variability per se but are an exciting new development in the analysis of mood data.

### Statement of conflicts of interest (bottom section of Table 1)

The field of ‘mHealth' (the delivery of healthcare services via mobile communication devices) is opaque and as in any other scientific field potential unstated economic and or scientific conflicts of interest may exist in studies. It is very important to disclose all potential conflicts, so the readers can evaluate the literature in an informed and proportionate way.

#### Ethics of digitally collected data

The use of digitally collected data for medical research poses unprecedented ethical challenges and should be considered and addressed during all phases of handling electronic data in research. These include questions related to individual privacy, data confidentiality, informed consent, involvement of commercial organizations, reuse of data, re-identification of de-identified data, differences in international privacy regulations, and changing societal attitudes towards public and private data^75^. Electronically collected data projects are often distributed across multiple countries, making issues of data management, privacy, and consent more complex. Cloud storage in unknown countries complicates legal jurisdiction. Privacy laws vary from country to country, and many countries have not addressed the impact of modern technology on existing regulations. In addition, there are many problems related to data created by patients. Patients may incorrectly assume that all medical privacy laws apply to commercial Internet companies, downloaded health apps, or data provided to health websites^75^.

## Conclusion

The present paper provides an overview of remotely collected electronic mood data in mood disorders research, discusses why standardized reporting is necessary to evaluate and advance research in Psychiatry, and presents reporting guidelines for remotely collected electronic mood monitoring. Adherence to these guidelines, including addressing ethical aspect of digitally collected data, will improve interpretation, reproducibility and facilitate future secondary analyses and meta-analyses of electronically collected mood data from independent studies.
